# Intelligent Informatics in Translational Medicine

**DOI:** 10.1155/2015/717210

**Published:** 2015-05-06

**Authors:** Hao-Teng Chang, Tatsuya Akutsu, Sorin Draghici, Oliver Ray, Tun-Wen Pai

**Affiliations:** ^1^Graduate Institute of Basic Medical Science, College of Medicine, China Medical University, Taichung 40402, Taiwan; ^2^Department of Computer Science and Information Engineering, Asia University, Taichung 41354, Taiwan; ^3^Department of Science Education, Affiliated Dongyang Hospital of Wenzhou Medical University, Dongyang, Zhejiang 322100, China; ^4^Bioinformatics Center, Institute for Chemical Research, Kyoto University, Kyoto 606-8501, Japan; ^5^Department of Computer Science, Wayne State University, Detroit, MI 48202, USA; ^6^Department of Computer Science, University of Bristol, Bristol BS8 1UB, UK; ^7^Department of Computer Science and Engineering, National Taiwan Ocean University, Keelung 20224, Taiwan; ^8^Center of Excellence for the Oceans, National Taiwan Ocean University, Keelung 20224, Taiwan

Starting from 2008, the organization committee members, Dr. Hui-Huang Hsu, Dr. Tun-Wen Pai, Dr. Oliver Ray, and Dr. Hao-Teng Chang, yearly held the International Workshop on Intelligent Informatics in Biology and Medicine (IIBM), which is used to be held in conjugation with International Conference on Complex, Intelligent, and Software Intensive Systems (CISIS). The main purpose for establishing this forum is to gather scientists from multidisciplinary fields including biology, medicine, computer science, statistics, and informatics to discuss how to face the new big data era and how to employ the hundreds of thousands of biological datasets including gene expression profiles, genetic information, proteomes, metabolomes, and even molecular imaging in clinical medicine. So many genomes from various species have been sequenced in quick succession. One important reason to conduct these genome projects is to translate useful and relevant information to biomedical research as well as therapeutic strategy development. From bench to bedside, bioinformatics researches have been provided strong and powerful tools to accelerate the analyses of complicated datasets.

In the past seven years, IIBM successfully brought together computer scientists, medical doctors, biologists, statisticians, and chemists to present and discuss current topics on genomics, epigenomics, GWAS/PheWAS, imaging processing, healthcare information, big data analyses, and so on. On the platform of IIBM, the scientists share their know-how and research experiences on processing the complexity and volume of experimental data from next generation sequencing and mass spectrometry technologies. Many various sophisticated computational methodologies have been designed and developed to support the new detection techniques which can effectively improve the quality of healthcare. Intelligent information technologies indeed facilitate and accelerate researches from basic to clinical investigation in terms of translational medicine.

To record the discoveries of talents and gather more contributions to these fields, this special issue was launched and supported by this journal. This special issue focuses on addressing the questions of biomedical sciences through computational technologies. It also describes the information processes employed, with an emphasis on forthcoming high throughput technologies and biomedical systems, which provides opportunities to discuss recent hot topics and progresses in the area of biomedicine for the academic and industrial societies. This special issue received 19 submissions from which 9 papers were selected for publication. These papers address the data-analytical method design, algorithm development, mathematical modeling, and computational simulation techniques of some translational medical applications.

In “Computational Biophysical, Biochemical, and Evolutionary Signature of Human R-Spondin Family Proteins, the Member of Canonical Wnt/ß-Catenin Signaling Pathway,” A. R. Sharma et al. applied biophysical, biochemical, and molecular evolutionary approaches to investigate human R-spondins protein family which is involved in cell growth and disease development and has been noticed as a potential therapeutic target. This bioinformatics study could be applied to the further investigation of Wnt/ß-catenin-System.

In “Detecting Epistatic Interactions in Metagenome-Wide Association Studies by metaBOOST,” M. Wu and R. Jiang proposed a method called metaBOOST to detect epistatic interactions between such metagenomic biomarkers as microbial genus and high-level functional KEGG orthologs. They performed comprehensive simulations to evaluate metaBOOST and applied the method to analyze two real genome-wide datasets for pathological mechanisms of microbial communities in human complex diseases.

In “Predicting Flavin and Nicotinamide Adenine Dinucleotide-Binding Sites in Proteins Using the Fragment Transformation Method,” C.-H. Lu et al. utilized fragment transformation methods to predict flavin and nicotinamide adenine dinucleotide-binding sites. The proposed method presents 68.4% and 67.1% true-positive rates for FAD and NAD binding site prediction under the false-positive rate at 5%, employing BioLiP dataset.

In “A Survey on the Computational Approaches to Identify Drug Targets in the Postgenomic Era,” Y.-F. Dai and X.-M. Zhao made a survey on the recent progress being made on computational methodologies that have been developed to predict drug targets based on different kinds of omics data and drug properties. This information could be utilized to improve prediction accuracy when developing new methodologies in the future.

In “Identification of Gene and MicroRNA Signatures for Oral Cancer Developed from Oral Leukoplakia,” G. Zhu et al. presented a new pipeline to identify oral cancer related genes and microRNAs by integrating both gene and miRNA expression profiles. They found some network modules as well as their miRNA regulators that play important roles in the development of oral leukoplakia to oral cancer. Among these network modules, 91.67% of genes and 37.5% of miRNAs have been previously reported to be related to oral cancer in literature.

In “A Heparan Sulfate-Binding Cell Penetrating Peptide for Tumor Targeting and Migration Inhibition,” C.-J. Chen et al. analyzed a set of heparan sulfate-binding cell penetrating peptides derived from natural proteins. In addition to cellular binding and internalization, these peptides demonstrated multiple functions including strong binding activities to tumor cell surface, significant inhibitory effects on cancer cell migration, and suppression of angiogenesis* in vitro* and* in vivo*.

In “A Large-Scale Structural Classification of Antimicrobial Peptides,” C.-C. Lee et al. presented a database of antimicrobial peptides (abbreviated as ADAM) which contains 7,007 unique sequences and 759 structures. ADAM systematically establishes comprehensive associations between AMP sequences and structures through structural folds and provides an easy access to view their relationships. Thirty distinct AMP structural fold clusters were detected and reported. According to ADAM, AMP structural folds are limited, only covering about 3% of the overall protein fold space.

In “Predict Metabolic Gene Biomarkers for Neurodegenerative Disease by an Integrated Network-Based Approach,” W. Tian et al. utilized Met-express method to predict key enzyme-coding genes in both Parkinson's and Huntington diseases. They found that the predicted genes might be involved in some common pathogenic metabolic pathways and had significant functional association with known disease genes. The predicted genes could be used as novel biomarkers for potential therapeutic treatments.

In “The TF-miRNA Coregulation Network in Oral Lichen Planus,” Y.-L. Zuo et al. employed the gene regulatory networks derived from transcriptomic and miRNA datasets to identify OLP related gene modules. In particular, they found that the gene modules were regulated by both transcription factors and miRNAs played important roles in the pathogenesis of OLP. Some of the genes in the modules have been reported to be related to the disease.

This special issue presents a broad spectrum of computational methodologies, biological investigation, and bioinformatics prediction. The papers included in this special issue provide useful messages of intelligent informatics for translational medicine applications. This issue illustrates that bioinformatics approaches can often be used by life scientists as a first step in the investigation of various disease mechanisms.

